# A Study of Dose Rate Probes for the País Vasco Environmental Radioactivity Automatic Network

**DOI:** 10.3390/s25216616

**Published:** 2025-10-28

**Authors:** Natalia Alegría, Miguel Angel Hernández-Ceballos, Igor Peñalva, Andima Freire, Jose Miguel Muñoz

**Affiliations:** 1 Energy Engineering Department, University of the Basque Country, 48013 Bilbao, Spain; igor.penalva@ehu.eus (I.P.); andima.freire@ehu.eus (A.F.); 2 Department of Physics, University of Cordoba, 14071 Córdoba, Spain; f92hecem@uco.es; 3 Department of Directorate of Industrial Development and Administration, Basque Government, 01003 Vitoria, Spain; josemi-munoz@euskadi.eus

**Keywords:** probes, environmental network, radioactivity

## Abstract

There are many types of probes available on the market for measuring ambient dose equivalent rates (ADERs), which makes intercomparison exercises essential to ensure data comparability and reliability. This study evaluated the performance of four widely used and similarly priced probes—the Reuter-Stokes ionization chamber, the RX04L from BITT, the MIRA from ENVINET, and the LB9360 from Berthold. The Reuter-Stokes ionization chamber was also taken as reference. Measurements were continuously conducted in Bilbao, northern Spain, during the period 2017–2021 under background conditions as well as during episodes of heavy rainfall and extreme temperatures. Results show that the BITT proportional counter exhibited the highest consistency with the Reuter-Stokes chamber under all meteorological conditions, and excellent stability even during extreme conditions. The Berthold probe displayed similar trends, but systematically overestimated dose rates, while the Geiger–Müller-based detector showed acceptable agreement under rainfall, but clear instability during temperature extremes. These findings highlight the importance of probe selection in environmental radioactivity networks as well as the use of reliable instruments for integration into modernized radiological surveillance systems.

## 1. Introduction

The Chernobyl nuclear accident in 1986 led to the establishment of radiological early warning networks across Europe under Council Decision 87/600/Euratom [1]. These systems were designed to ensure the rapid detection and quantification of radioactive releases into the environment. Key initiatives include the European Radiological Data Exchange Platform (EURDEP) [2], which enables international data sharing [3], and the International Monitoring System (IMS) [4] of the Comprehensive Nuclear-Test-Ban Treaty Organization (CTBTO), which tracks radioactive aerosols and noble gases worldwide [5].

In Spain, this effort resulted in the creation of the national *Red de Estaciones Automáticas* (REA) [6] of the Nuclear Safety Council (CSN) [7]. Within this framework, the Basque Country established its own radiological surveillance infrastructure (RAREx) in 2001, in collaboration with the University of the Basque Country (UPV/EHU) and the Basque Government [8]. Initially equipped with proportional counters, the network is currently undergoing modernization due to detector aging and the availability of new technologies. One of its main objectives is to provide accurate real-time measurements of the ambient dose equivalent rate, H*(10), an internationally endorsed parameter essential for reliable decision-making during radiological emergencies.

Similar challenges are faced by other European monitoring networks, which must balance cost, robustness, and measurement accuracy. For instance, Germany’s ODL network [9] operates more than 1800 automatic dose rate stations and has studied meteorological influences on its data. In the Netherlands, rainfall radar analyses revealed strong correlations between rainfall intensity and short-term dose rate increases due to radon progeny washout [10]. Likewise, studies in Romania highlighted the role of radon progeny and weather variables in shaping ambient dose rate measurements [11]. These experiences underscore the importance of intercomparison exercises and careful instrument selection to ensure the reliability and comparability of monitoring systems.

Meteorological effects are particularly relevant in regions with highly variable weather. For instance, meteorological conditions influence the attenuation of gamma rays (via humidity) and Rn exhalation from soil, which contributes significantly to dose rate fluctuations [12]. Rainfall enhances the deposition of natural radionuclides such as ^7^Be, ^210^Pb, ^40^K, and ^222^Rn, often producing transient dose rate peaks, while long dry spells followed by rainfall can further amplify these effects [13]. Temperature extremes may also induce calibration drifts in detectors based on Geiger–Müller tubes, altering counting efficiency and stability [14]. High humidity and variations in atmospheric pressure also influence radon exhalation from soils and the airborne concentration of its progeny [15]. Given the humid oceanic climate of the Basque Country, with frequent rainfall and moderate but variable temperatures, assessing detector performance under such conditions is particularly relevant. In addition, snow and wind are two meteorological parameters that influence dose rate values [16,17].

This paper presents a comparative analysis of four dose rate probes—the RX04L (BITT) [18], the MIRA (ENVINET) [19], the Reuter-Stokes ionization chamber (Westinghouse) [20], and the LB9360 (Berthold) [21]. Measurements were evaluated against the Reuter-Stokes ionization chamber under different meteorological conditions, including heavy rainfall and extreme temperatures, to determine the robustness, sensitivity, and reliability of each probe for potential integration into the upgraded RAREx network.

## 2. Materials and Methods

### 2.1. Meteorological Characteristics of the Monitoring Site

**Figure 1:**
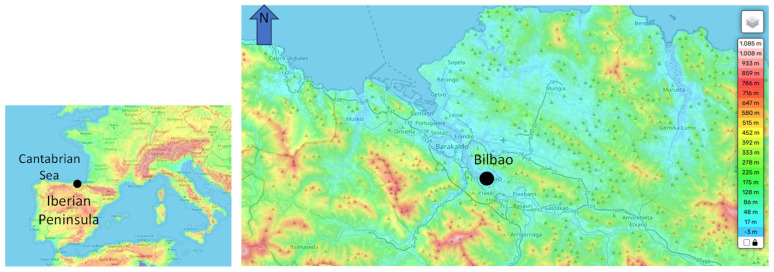
Location of the study site on the Iberian Peninsula and topographic map of the surroundings of Bilbao.

The comparison was carried out in Bilbao (Figure 1), the largest city of the País Vasco in northern Spain, located in the Nervión Valley about 16 km from the Bay of Biscay [22]. The region has a temperate oceanic climate dominated by Atlantic air masses, characterized by mild winters (average 6–7 °C on the coast), warm but moderate summers (25–26 °C, occasionally >30 °C inland), and high relative humidity (70–80%). Annual rainfall usually exceeds 1200 mm, with rainfall distributed throughout the year and frequent cloudy conditions. The local topography, consisting of low mountain ranges (1000–1500 m) and narrow valleys, further influences wind circulation and rainfall patterns.

### 2.2. Description of Radiation Monitoring Probes

**Figure 2:**
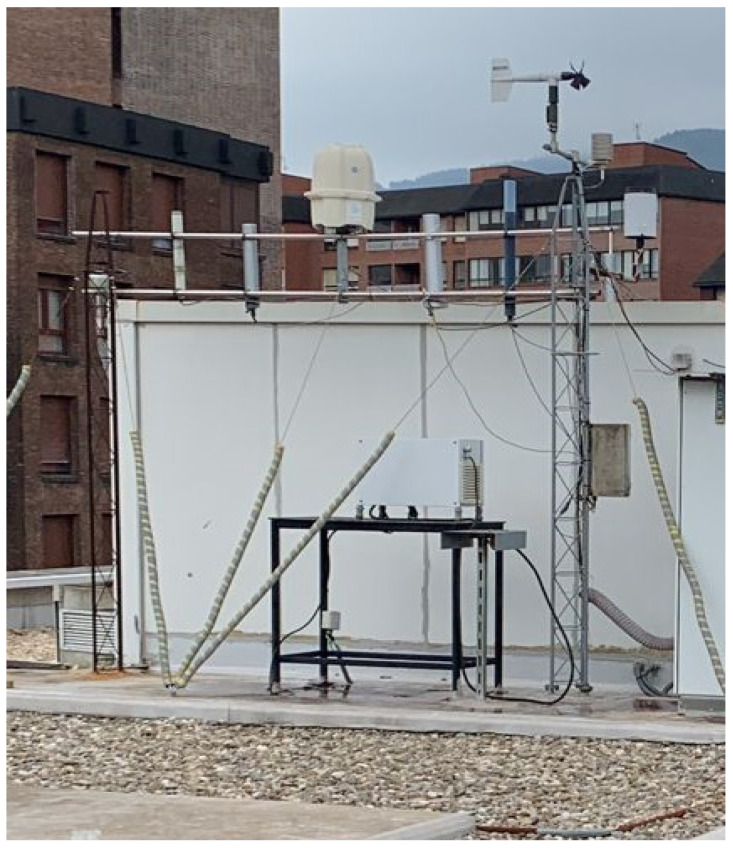
Radiation monitoring probes and meteorological station installed on the rooftop of the School of Engineering in Bilbao.

All detectors were installed in 2015 on the rooftop of the School of Engineering in Bilbao (Figure 2). The experimental setup included a Reuter-Stokes ionization chamber (Westinghouse), two proportional counters (the RX04L from BITT and the LB9360 from Berthold), and one Geiger–Müller detector (the MIRA from ENVINET). Each probe recorded values every ten minutes. The Reuter-Stokes chamber, considered the most accurate, but also the most expensive, option, was used as the reference instrument throughout the comparison. To validate the manufacturer’s specifications, additional comparison exercises were conducted at PTB Braunschweig [23]. At PTB, a comparison exercise determined the intrinsic background and cosmic radiation contribution, and validated the system’s response through a simulated radioactive plume involving Co-60, Cs-137, and Ra-226 sources. In addition, these four dose rate probes were calibrated annually using a Cs-137 reference source. The coefficient factor obtained after calibration, which is applied before performing all measurements, is one for all these probes except the Berthold probe. Berthold probes have a calibration factor generic of 0.122 µSv/h per cps [24].

The RX04L (BITT) is a compact proportional counter designed for continuous outdoor operation. It measures dose rates from tens of nSv/h up to several mSv/h and incorporates sensors for temperature, humidity, and pressure. Its communication interfaces (GPRS, RS232, Ethernet) facilitate integration into automatic monitoring networks, while its weatherproof housing ensures resistance to environmental stress. The LB9360 (Berthold) is another proportional counter widely used in European radiological networks. It covers a dose rate range of 10 nSv/h to 10 Sv/h and includes built-in environmental sensors for automatic correction of meteorological effects. Its main strengths are long-term stability and low maintenance requirements, although previous evaluations have reported a systematic tendency to overestimate dose rates compared with ionization chambers. The Geiger–Müller-based probe was the MIRA (ENVINET), which covers wide measurement ranges (from a few nSv/h up to several Sv/h) and is optimized for large-scale surveillance networks. The MIRA probe incorporates electronic compensation algorithms to mitigate the influence of temperature and humidity. Nonetheless, GM detectors are generally more sensitive to environmental variability, particularly temperature extremes.

In 2022, the probes were removed due to several problems with electrical connections. In 2025, the probes were placed in the first location and started again.

### 2.3. Meteorological Data

A Campbell Scientific weather station was also installed on the rooftop of the School of Engineering in Bilbao (Figure 2). It recorded meteorological parameters every ten minutes, including air temperature (°C) and rainfall (mm). This configuration enabled the simultaneous monitoring of dose rates and environmental variables, providing a robust framework for evaluating detector performance under real atmospheric conditions. Temperature and rainfall were selected as the key meteorological variables for analysis because they are known to exert the strongest influence on ambient dose rate measurements. Temperature variations can affect the electronic stability and calibration of detectors, particularly those based on Geiger–Müller tubes, leading to shifts in their response under extreme conditions. Rainfall, on the other hand, strongly influences the washout and deposition of radon progeny and cosmogenic radionuclides, often producing transient increases in dose rate values.

### 2.4. Statistical Analysis

The relationship between variables was evaluated using the Pearson correlation coefficient (*r*) and the coefficient of determination (*R*^2^). The Pearson coefficient quantifies the strength and direction of the linear relationship between two variables and is defined as

$$r=\frac{\sum_{i=1}^{N}\left(x_{i}-\bar{x}\right)\left(y_{i}-\bar{y}\right)}{\sqrt{\sum_{i=1}^{N}{\left(x_{i}-\bar{x}\right)}^{2}\sum_{i=1}^{N}{\left(y_{i}-\bar{y}\right)}^{2}}}$$

It ranges between −1 and +1, where values close to +1 indicate a strong positive linear correlation, values close to −1 indicate a strong negative linear correlation, and values near 0 suggest the absence of a linear relationship.

The coefficient of determination (*R*^2^) expresses the proportion of the variance in the dependent variable that is predictable from the independent variable and is given by

$$R^{2}=1-\frac{\sum_{i=1}^{N}{\left(y_{i}-\hat{y_{i}}\right)}^{2}}{\sum_{i=1}^{N}{\left(y-\bar{y}\right)}^{2}}$$

It ranges from 0 to 1, with higher values indicating that a greater proportion of the variance in the dependent variable is explained by the independent variable.

In addition, simple linear regression was applied to quantify the linear dependence and to model the relationship between analyzed parameters. These statistical tools provided a robust quantitative framework for assessing the consistency, strength, and reliability of the observed associations.

Finally, seasons were defined as winter, January to March; spring, April to June; summer, July to September; and autumn, October to December.

## 3. Results and Discussion

### 3.1. ADER and Meteorological Data (2017–2021)

**Figure 3:**
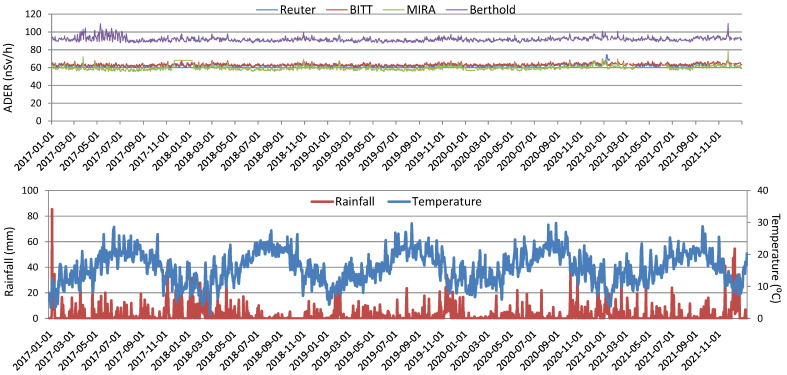
Daily evolution of ADER, temperature, and rainfall data during the period 2017–2021 in Bilbao.

Figure 3 shows the daily evolution of the ambient dose equivalent rate (ADER) together with temperature and rainfall for the period 2017–2021. The proportion of valid daily records for each sensor exceeded 90% for both ADER and meteorological data, ensuring the robustness and representativeness of the dataset. Average ADER values during this period were 62.402 ± 0.037 nSv/h (Reuter-Stokes), 63.473 ± 0.032 nSv/h (BITT), 59.676 ± 0.062 nSv/h (MIRA), and 99.545 ± 0.058 nSv/h (Berthold), in which the uncertainty of each is provided as the standard error of the mean (Sx/N^1/2^, Sx being the standard deviation and N the number of values summarized in the average), which corresponds to a coverage factor of k = 1. The mean daily temperature over the study period was 15.20 ± 0.13 °C, while cumulative rainfall reached 4893.0 mm, with a maximum daily value of 85.3 mm. Notably, days with rainfall (886 days with rainfall above 0 mm) were similar to days without rainfall (940 days), underlining the importance of rainfall as a key environmental factor influencing ADER measurements in Bilbao.

### 3.2. Correlation Between Dosimetric Data

At first inspection, all probes reproduced the general temporal evolution of ADER values. This observation was confirmed by correlation analyses (Table 1), with coefficients ranging from 0.94 (Reuter-Stokes –BITT) to 0.74 (Berthold–MIRA). All values were statistically significant at the 95% confidence level, indicating that despite systematic differences, all instruments captured the main temporal trends. When correlations with the Reuter-Stokes ionization chamber were considered, coefficients consistently exceeded 0.77. The strongest agreement was observed for the BITT proportional counter (*r* = 0.94), while the Berthold probe showed moderate correlation (*r* = 0.83), but with an evident overestimation bias.

**Figure 4:**
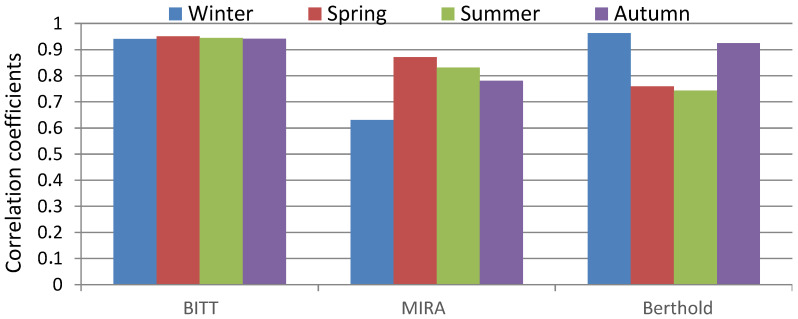
Seasonal variability of correlation coefficients of daily values during the period 2017–2021 between Reuter-Stokes and BITT, MIRA, and Berthold probes.

Seasonal correlations (Figure 4) reveal additional variability. The BITT probe maintained consistently high correlations (>0.90) across all seasons, confirming its stability. The MIRA detector showed good performance in spring (*r* = 0.88), but weaker in winter (*r* = 0.63), suggesting sensitivity to low temperatures. The Berthold probe correlated well in winter and autumn (>0.90), but its performance decreased in spring and summer (<0.75), reflecting seasonal instability. These findings indicate that while overall correlations are high, environmental conditions modulate detector performance differently depending on the probe type.

### 3.3. Dependence of Temperature and Rainfall

**Figure 5:**
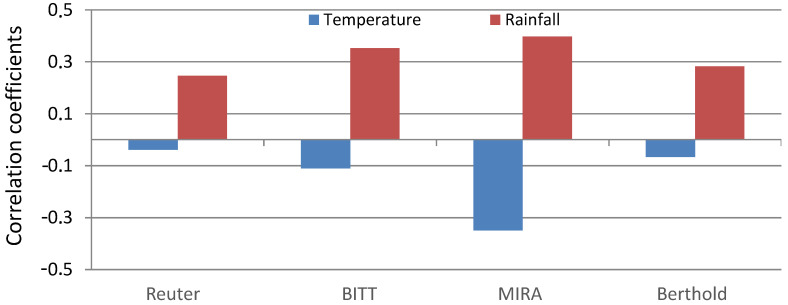
Correlation coefficients of daily values between ADER in Reuter-Stokes, BITT, MIRA, and Berthold probes and temperature and rainfall.

Correlations between ADER and meteorological variables (Figure 5) highlight the different impacts of temperature and rainfall. Temperature shows weak negative correlations with all probes (−0.03 for Reuter-Stokes to −0.35 for MIRA), suggesting limited but systematic influence. Rainfall exhibits stronger positive correlations (0.28 for Reuter-Stokes to 0.40 for MIRA), consistent with the washout and deposition of radon progeny and cosmogenic radionuclides during rainfall events.

**Figure 6:**
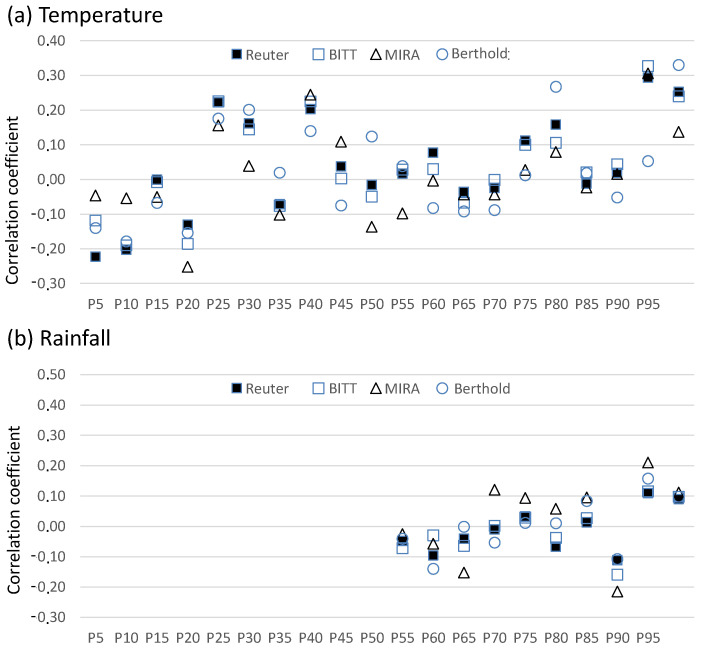
Variability of correlation coefficients between ADER concentrations and (**a**) temperature and (**b**) rainfall in different ranges (percentiles).

Percentile-based analysis (Figure 6) provides further insights. For temperature, correlations are generally weak and variable, with a tendency toward positive values at the highest percentiles. For rainfall, correlations remain weak at low-to-intermediate percentiles, but strengthen under wetter conditions (>P90), with a consistent positive correlation across all probes at the highest percentile. These results confirm that both temperature and rainfall exert only moderate effects under normal conditions, but their influence becomes more pronounced during extreme events.

**Figure 7:**
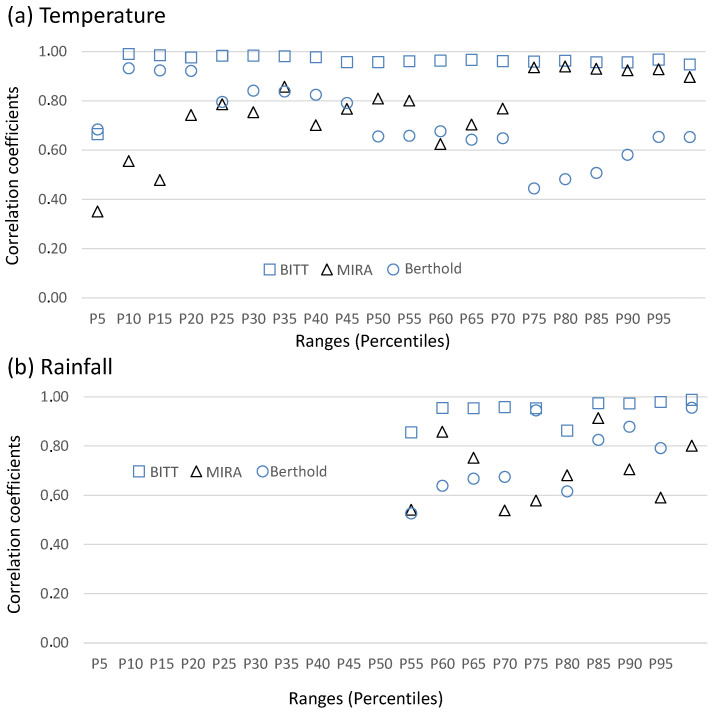
Variability of correlation coefficients of ADER concentrations between Reuter-Stokes and BITT, MIRA, and Berthold probes for different ranges (percentiles) in (**a**) temperature and (**b**) rainfall.

Figure 7 compares probe–Reuter-Stokes correlations across percentiles of temperature and rainfall. The BITT probe consistently shows the highest agreement with the Reuter-Stokes chamber, with correlations approaching unity even under variable conditions in temperature and rainfall. The MIRA generally performed well, especially at the highest temperatures, but shows fluctuations, particularly at low temperature levels, with correlations below 0.6. In the case of rainfall, it shows large variability, but with correlation coefficients above 0.5. The Berthold probe displays marked variability, with high correlation coefficients at low temperature levels, falling below 0.6 under certain temperature ranges, underscoring its reduced reliability under high temperature conditions. In the case of rainfall, the correlation is always above 0.6, reaching the maximum in the highest amount of rainfall.

### 3.4. Behaviour Under Extreme Rainfall and Temperature Events

**Figure 8:**
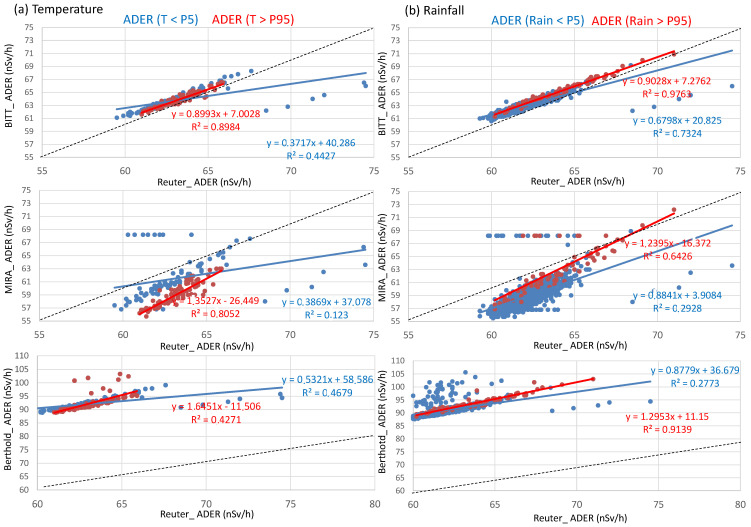
Scatter plot between ADER values of Reuter-Stokes and BITT, MIRA, and Berthold probes under extreme daily values of temperatures and rainfall (below 5th percentile and above 95th percentile).

Figure 8 summarizes the performance of the probes under extreme meteorological conditions, defined as values below the 5th percentile and above the 95th percentile for both temperature and rainfall. Under the highest temperatures the BITT probe exhibited the best agreement with the Reuter-Stokes reference, with a slope close to unity (0.88) and a high coefficient of determination (*R*^2^ > 0.89). On the contrary, under the lowest temperatures, it showed a lower slope (0.37) and smaller *R*^2^ (0.44), indicating a biased response between both cold and hot conditions. The MIRA detector showed reasonable agreement at high temperatures (*R*^2^ ≈ 0.80; slope ≈ 1.35), but under cold conditions the response deteriorated substantially (*R*^2^ ≈ 0.12; slope ≈ 0.38), suggesting an underestimation of the measured dose rate and higher variability. In contrast, the Berthold probe displayed the most pronounced deviations: during high and low temperature periods, the slopes reached 1.64 and 0.53, respectively, although with high coefficients of determination (*R*^2^ > 0.42).

Under rainfall extremes, similar trends are observed. The BITT probe maintained the most consistent behavior, with slopes near unity (0.90 and 0.68) and excellent linearity (*R*^2^ ≥ 0.97 during heavy rainfall), confirming its robustness under both dry and wet conditions. The MIRA detector achieved moderate consistency under high rainfall (*R*^2^ ≈ 0.64; slope ≈ 1.24), although it underperformed in dry conditions (*R*^2^ ≈ 0.29; slope ≈ 0.88), indicating a limited sensitivity range. Again, the Berthold probe showed significant discrepancies: despite a strong correlation under heavy rain (*R*^2^ ≈ 0.91; slope ≈ 1.30), the positive intercept (+11.15) reveals a systematic offset that may lead to overestimation of ambient dose rates, whereas under dry conditions (*R*^2^ ≈ 0.28) its performance became unstable and less reliable.

These results confirm the robustness of the BITT proportional counter across diverse environmental scenarios, the moderate reliability, but temperature sensitivity, of the MIRA detector, and the systematic overestimation and instability of the Berthold probe, especially under extreme weather conditions.

## 4. Conclusions

Comparative analysis of three automatic probes for ambient dose equivalent rate measurements against a Reuter-Stokes ionization chamber during the period 2017–2021 demonstrated that probe performance is strongly conditioned by meteorological variability, especially under extreme temperature and rainfall events. The BITT RX04L proportional counter consistently reproduced the reference values with high accuracy and stability, confirming its robustness as a reliable alternative for long-term monitoring. The Berthold LB9360 also showed a coherent temporal evolution, but systematically overestimated the measured dose rates, which may compromise its usefulness in networks that require precise absolute values. By contrast, the Geiger–Müller-based probe exhibited greater sensitivity to environmental changes: while its response to rainfall events was reasonable, its instability at temperature extremes limits its reliability for continuous surveillance. Overall, this study underlines the necessity of performing comparison exercises prior to the deployment of detectors in environmental radioactivity networks and provides empirical evidence that supports the integration of proportional counters.

## Figures and Tables

**Table 1:** Correlation coefficient between daily ADER values of different probes. All are statistically significant at 95%.

	Reuter-Stokes	BITT	MIRA	Berthold
Reuter-Stokes	1.00	0.94	0.77	0.83
BITT		1.00	0.85	0.86
MIRA			1.00	0.74
Berthold				1.00

## Data Availability

The original contributions presented in this study are included in the article. Further inquiries can be directed to the corresponding author.
